# Supplementary figures and images for: Follicular CD8+ T cells in Trypanosoma cruzi infection: helpers or killers depending on the target B cell population
Source: PLoS Pathog. 2025 Oct 17;21(10):e1013595. doi: 10.1371/journal.ppat.1013595 (PMC12558609; doi:10.1371/journal.ppat.1013595)

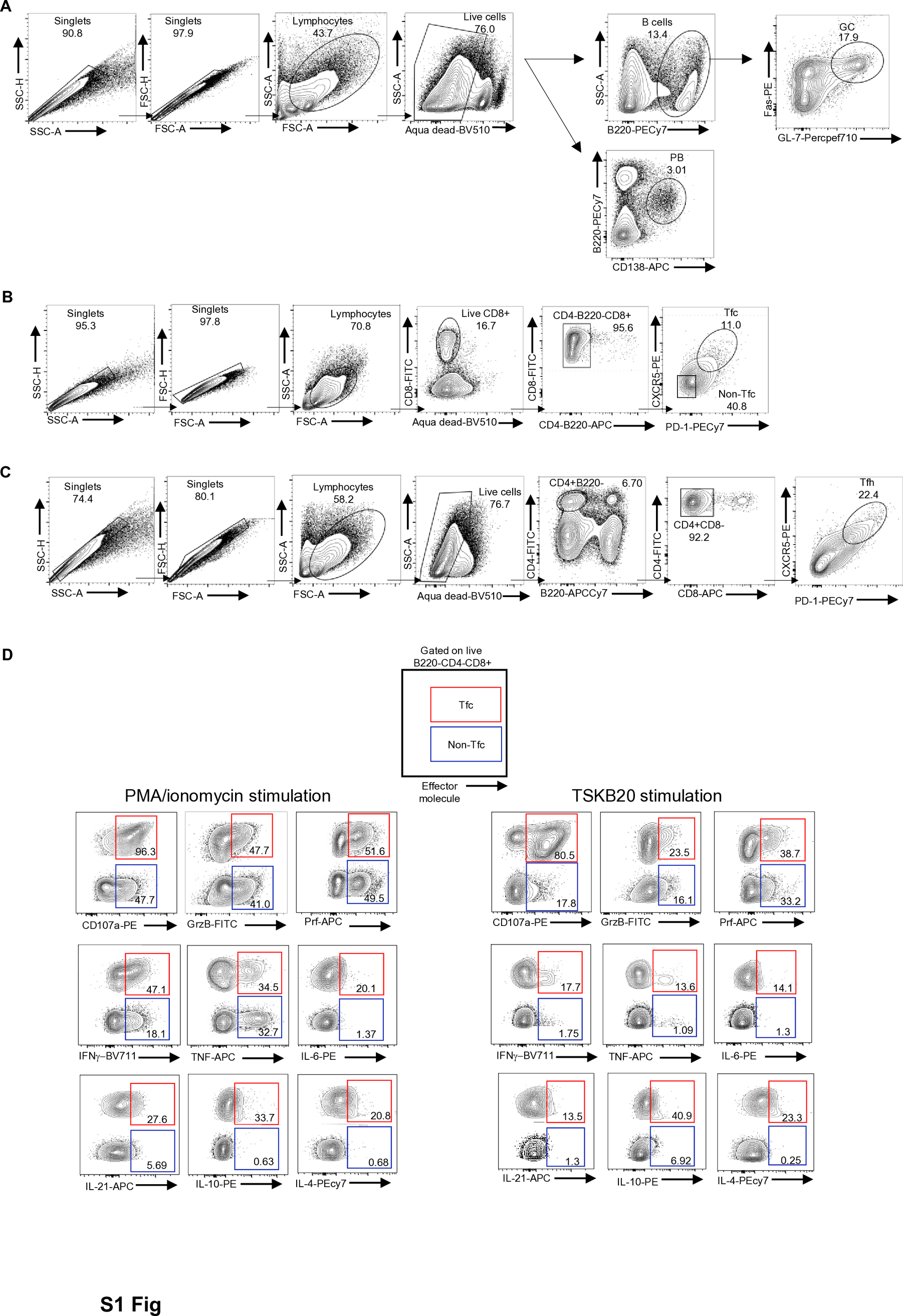

Supplement: S1 Fig — Representative flow cytometry contour plots illustrating gating strategies. After exclusion of doublets, lymphocytes were identified based on forward (FSC-A) and side scatter (SSC-A) parameters. Live cells were gated by excluding those stained with Live/Dead Fixable Aqua 405. (A) B cells were selected based on B220 expression. Within this population, GC B cells were identified based on Fas and GL-7 expression, while plasmablasts (PB) were identified within live lymphocytes based on B220int and CD138 expression. (B) Representative flow cytometry plots showing the gating strategy used to identify Tfc CD8 ⁺ T cells (CXCR5 ⁺ PD-1⁺) and Non-Tfc (CXCR5 ⁻ PD-1⁻) CD8 ⁺ T cells from the spleens of T. cruzi-infected mice. Gating was performed sequentially on singlets, live CD8 ⁺ cells (CD4 ⁻ B220⁻), and subsequently on CXCR5 vs. PD-1 expression. Percentages represent the proportion of cells within the respective parent gate. (C) Representative flow cytometry plots illustrating the gating strategy used to identify Tfh CD4 ⁺ T cells (CXCR5 ⁺ PD-1⁺) from the spleens of T. cruzi-infected mice. Gating was performed sequentially on singlets, live CD4 ⁺ cells (CD8 ⁻ B220⁻), followed by CXCR5 and PD-1 expression. Percentages indicate the proportion of cells within the respective parent gate. (D) Assessment of cytokine expression (IFN-γ, TNF, IL-6, IL-21, IL-10, IL-4), GrzB, Prf, and CD107a in Tfc (CXCR5 ⁺ PD-1⁺) and Non-Tfc (CXCR5 ⁻ PD-1⁻) CD8+ T cells following stimulation with either PMA/ionomycin or the specific peptide TSKB20. Each plot shows the frequency of Tfc or Non-Tfc CD8 ⁺ T cells expressing the indicated effector molecule. Percentages within the quadrants indicate the proportion of cytokine-positive cells within the respective gated population. (TIF) [file ppat.1013595.s001.tif]

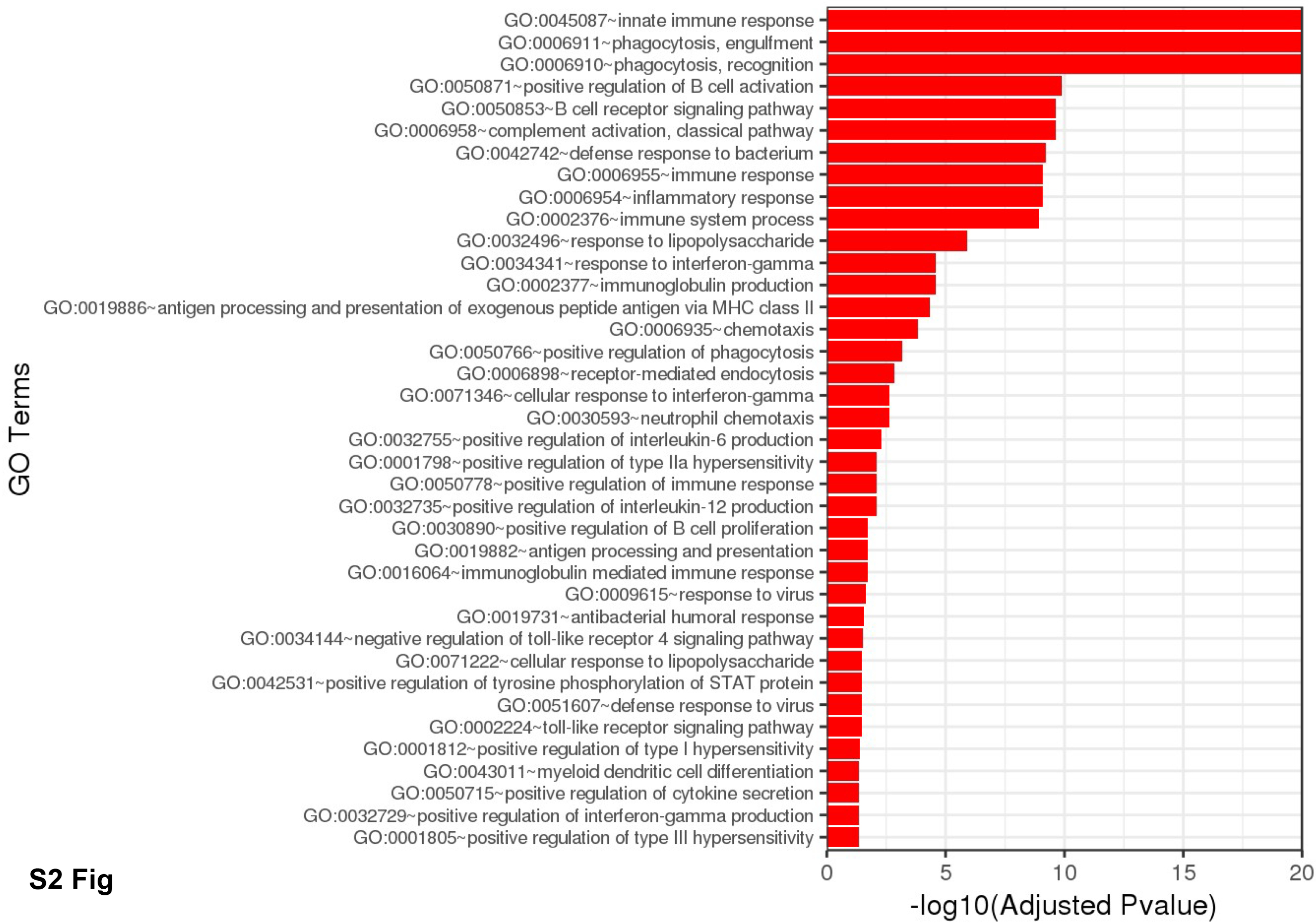

Supplement: S2 Fig — Gene Ontology (GO) enrichment analysis based on RNA-seq data from Tfc cells, respect to Non-Tfc cells. The plot displays up to 40 significantly enriched GO terms (adjusted p-value, -log10(adjusted p-value)), focusing on categories related to immune responses, cellular activation, and cytokine regulation. (TIF) [file ppat.1013595.s002.tif]

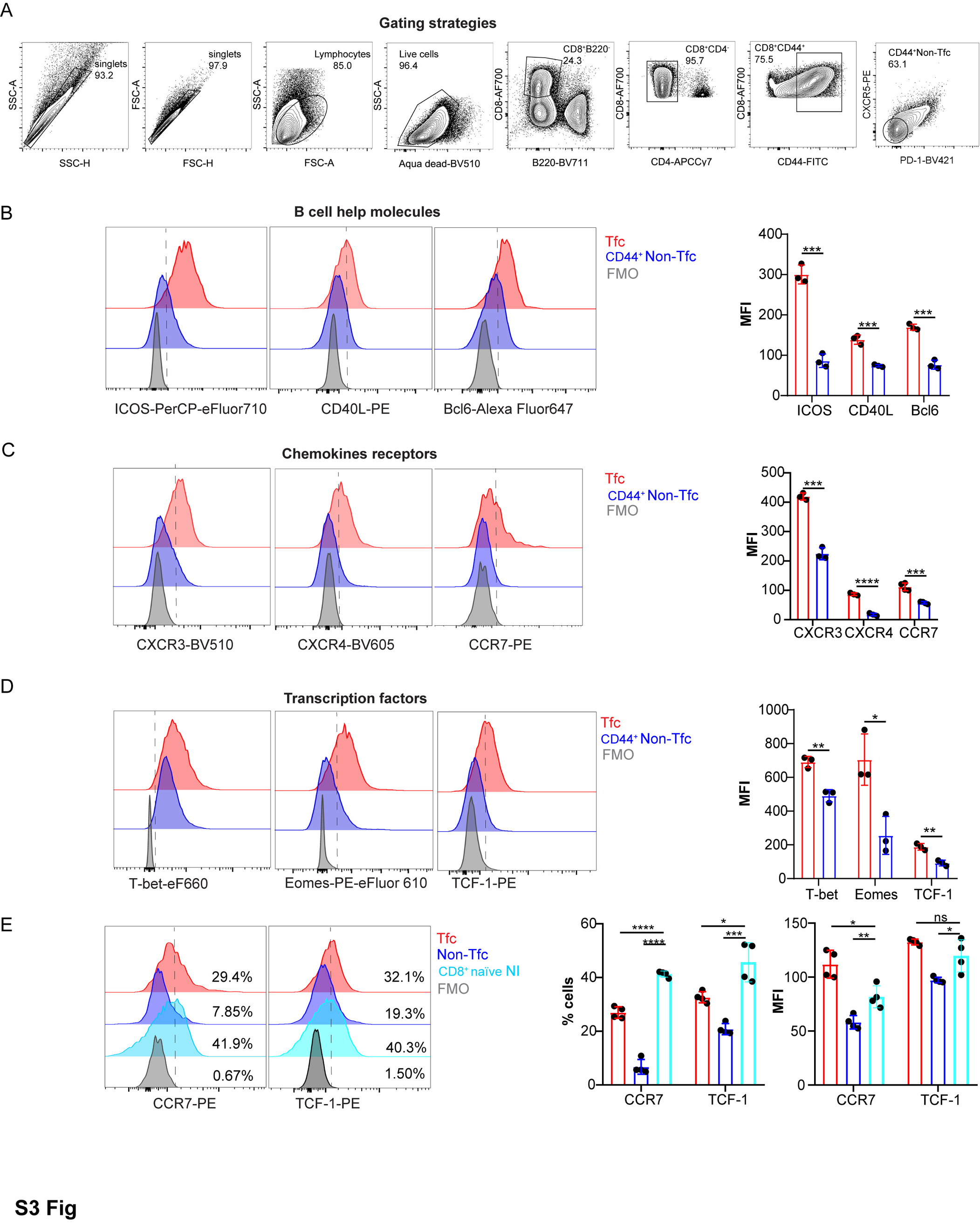

Supplement: S3 Fig — Splenic cells from non-infected and T. cruzi infected mice were collected at 18 dpi and analyzed by flow cytometry. (A) Representative contour plots showing the gating strategy used to identify CD44 ⁺ Non-Tfc (CXCR5 ⁻ PD-1 ⁻ CD8⁺) T cells. (B–D) Representative histograms and corresponding quantification of MFI for (B) B cell help-associated molecules (ICOS, CD40L, and Bcl6), (C) chemokine receptors (CXCR3, CXCR4, and CCR7), and (D) transcription factors (T-bet, Eomes, and TCF-1) in Tfc cells (all CD44⁺) and activated (CD44⁺) Non-Tfc CD8 ⁺ T cells. (E) Representative histograms and quantification of MFI and frequency of CCR7⁺ and TCF-1 ⁺ cells determined using FMO controls for Tfc and Non-Tfc cells (without excluding naïve T cells) from infected mice, and for naïve CD8 ⁺ T cells from non-infected mice (CD8 ⁺ naïve NI, light blue). Data are presented as mean ± SD; N = 3 (A-D) and N = 4 mice. Data were collected from 2 independent experiments. Statistical significance was determined by an unpaired t-test (A-D) and an unpaired t-test by comparing CD8+ naïve NI - Tfc and CD8+ naïve NI - Non-Tfc cells (*p < 0.05, **p < 0.01, ***p < 0.001, ****p < 0.0001). (TIF) [file ppat.1013595.s003.tif]

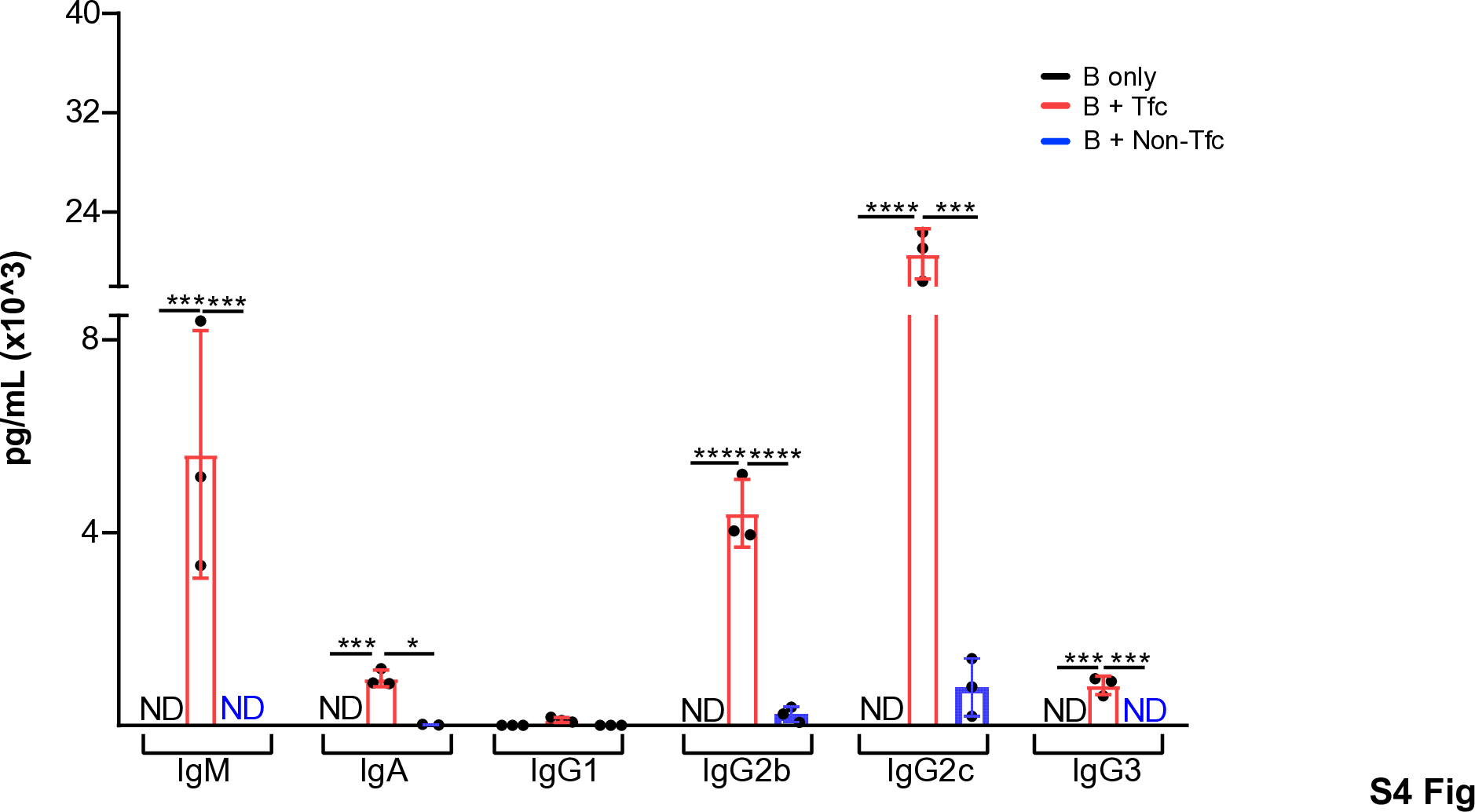

Supplement: S4 Fig — Naïve B cells from non-infected mice were co-cultured with either Tfc or Non-Tfc cells isolated from infected mice at 18 dpi. The graph shows the concentrations of Igs measured in the culture supernatants. ND: not detected. For statistical purposes the samples with ND values were imputed with a value equal to the lower detection limit of the technique employed. Data are presented as mean ± SD. Each symbol indicates an individual animal, N = 3. Data are representative of two independent experiments. Statistics: ordinary one-way ANOVA with select comparisons and a Bonferroni correction. *p < 0.05, ***p < 0.001, ****p < 0.0001. (TIF) [file ppat.1013595.s004.tif]

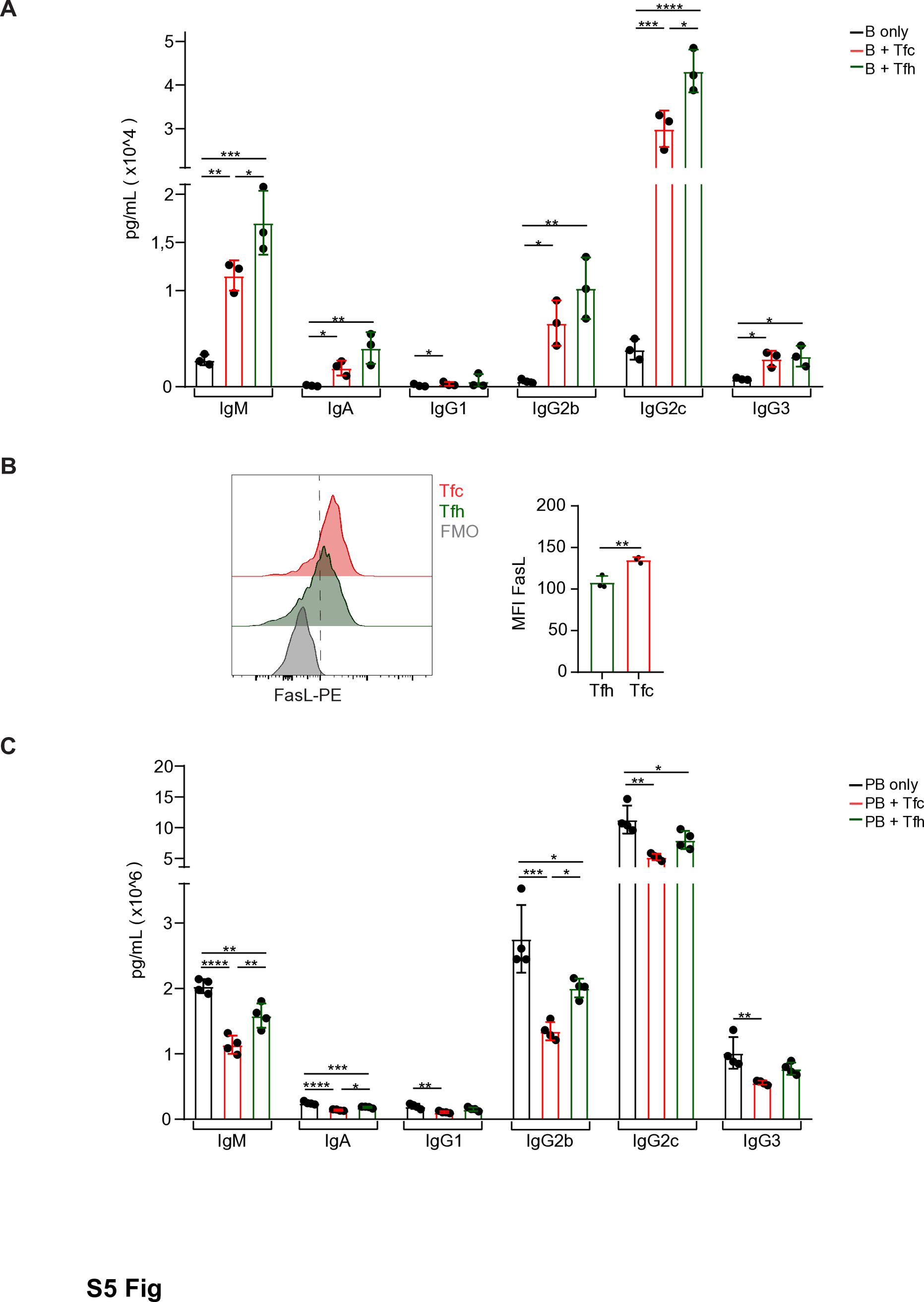

Supplement: S5 Fig — (A) Sorted naïve B cells or (C) plasmablasts were co-cultured for 20 h with either medium alone (black), sorted Tfc cells (red), or sorted Tfh cells (green), in the presence of anti-CD3 plus anti-CD28. All cell populations were purified from the spleens of T. cruzi-infected mice at 18 dpi. (A, C) Statistical analysis of Ig concentrations (IgM, IgA, IgG1, IgG2b, IgG2c, and IgG3) measured in the culture supernatants using a multiplex bead-based assay. (B) Representative histogram and statistical analysis of MFI of FasL. Data are presented as mean ± SD. Each dot represents an individual mouse, N = 3. (A-C) Data are representative of 2 independent experiments. Statistical analyses: ordinary one-way ANOVA with selected comparisons and Bonferroni correction (A, C); unpaired t-test (B). *p < 0.05, **p < 0.01, ***p < 0.001, ****p < 0.0001. (TIF) [file ppat.1013595.s005.tif]

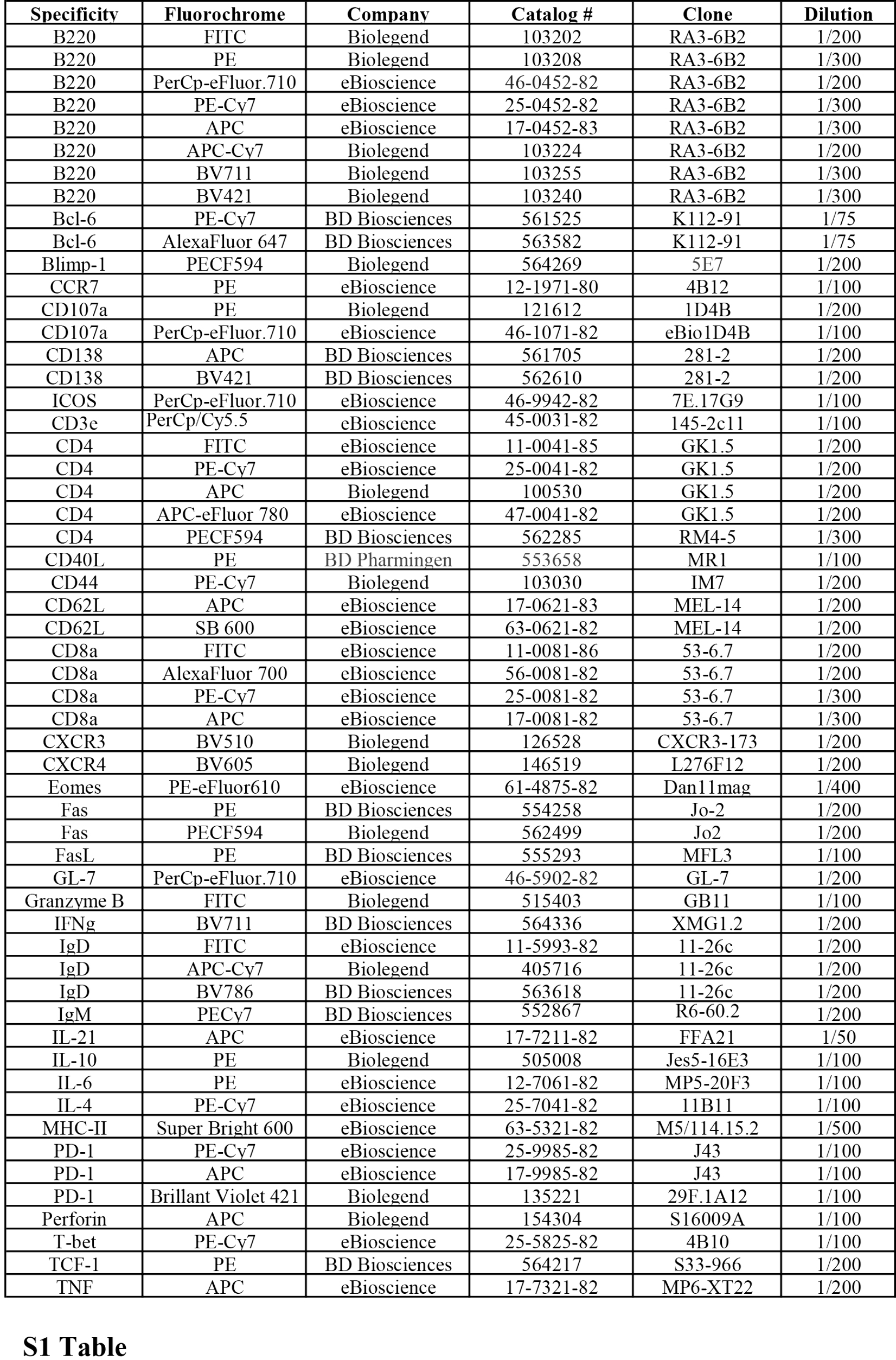

Supplement: S1 Table — Detailed list of Abs used in flow cytometry experiments, including their target specificity, fluorochrome conjugate, supplier, catalog number, clone, and working dilution. (TIF) [file ppat.1013595.s006.tif]
