# Supplementary material for: Follicular CD8+ T cells in Trypanosoma cruzi infection: helpers or killers depending on the target B cell population
Source: PLoS Pathog. 2025 Oct 17;21(10):e1013595. doi: 10.1371/journal.ppat.1013595 (PMC12558609; doi:10.1371/journal.ppat.1013595)
Supplement: Attachment — Submitted filename: Response to Reviewers.docx [file ppat.1013595.s008.docx]

Córdoba, Argentina, August 6th, 2025.-

Martin Craig Taylor
Guest Editor-PLOS Pathogens

Dominique Soldati-Favre
Section Editor-PLOS Pathogens

Sumita Bhaduri-McIntosh and Michael Malim
Editors-in-Chief-PLOS Pathogens

Thanks for your correspondence clarifying the basis of the Editorial Board´s decision about our manuscript PPATHOGENS-D-25-00863 “**Follicular CD8+ T cells in *Trypanosoma cruzi* infection: helpers or killers depending on the target B cell population**”. We are glad to know that the topic of the study is of interest to the PLOS Pathogens Editorial Board. We also would like to thank the reviewers for their thoughtful review of the manuscript, for constructive criticisms and valuable comments. They raised important issues, and their inputs were very helpful for improving the manuscript. We agree with almost all their comments, and we have revised our manuscript accordingly. We are including all reviewers’ suggestions and clarifying the text when needed. We are confident that the revised version of the manuscript is greatly improved.

We respond below in detail to each of the reviewer’s comments. In addition, we included the requested information that was incorporated into the text manuscript in different sections (results, material and methods, legends for figures and discussion). We hope that the reviewers will find our responses to their comments satisfactory. In addition, we have incorporated the information requested by the Journal Requirements.

Please, find below the referees’ comments repeated in italic and our responses inserted after each comment in red. To facilitate the work of the reviewers, in some instances we refer to the former manuscript indicating the page and the line (page-line).

Finally, we would like to highlight that this study describes the presence of follicular cytotoxic CD8⁺ T cells in *T. cruzi*-infected mice and their differential impact on distinct B cell populations. Tfc cells may contribute to the regulation of early antibody responses during *T. cruzi* infection by combining helper and cytotoxic functions. Tfc cells can act as helper cells on naïve and activated B cells and can kill highly differentiated B cells, as plasmablasts.

By the reasons discussed above, we are convinced that our findings do provide an important conceptual advance for the field and are of interest to the broad audience of PLOS Pathogens.

Looking forward to hearing from you soon,

Sincerely,

**Adriana Gruppi**

Centro de Investigaciones en Bioquímica Clínica e Inmunología.

Facultad de Ciencias Químicas. Universidad Nacional de Córdoba

**Journal Requirements:**

*1) Please ensure that the CRediT author contributions listed for every co-author are completed accurately and in full. At this stage, the following Authors/Authors require contributions: Yamila Gazzoni, Laura Almada, Julio Cesar Gareca, Carolina Lucia Montes, Eva Virginia Acosta-Rodriguez, and Adriana Gruppi. Please ensure that the full contributions of each author are acknowledged in the "Add/Edit/Remove Authors" section of our submission form.*

The full contributions of each author were indicated in the "Add/Edit/Remove Authors" section of the submission form provided by PLOS Pathogens.

*2) We ask that a manuscript source file is provided at Revision. Please upload your manuscript file as a .doc, .docx, .rtf or .tex. If you are providing a .tex file, please upload it under the item type LaTeX Source File and leave your .pdf version as the item type Manuscript.*

As requested, we have uploaded the manuscript source files (Revised Manuscript with track changes and “Manuscript”) in the required format (.doc).

*3) We do not publish any copyright or trademark symbols that usually accompany proprietary names, eg ©, ®, or TM (e.g. next to drug or reagent names). Therefore, please remove all instances of trademark/copyright symbols throughout the text.*

The symbol ® on page 28 and TM on pages 27, and 28 were removed.

*4) Some material included in your submission may be copyrighted. According to PLOS’s copyright policy, authors who use figures or other material (e.g., graphics, clipart, maps) from another author or copyright holder must demonstrate or obtain permission to publish this material under the Creative Commons Attribution 4.0 International (CC BY 4.0) License used by PLOS journals. Please closely review the details of PLOS’s copyright requirements here: PLOS Licenses and Copyright. If you need to request permissions from a copyright holder, you may use PLOS's Copyright Content Permission form.
Please respond directly to this email and provide any known details concerning your material's license terms and permissions required for reuse, even if you have not yet obtained copyright permissions or are unsure of your material's copyright compatibility. Once you have responded and addressed all other outstanding technical requirements, you may resubmit your manuscript within Editorial Manager. 
Potential Copyright Issues: i) Figures 5A, 5C, 5D, 5E, 6A, 7A, 7C, and 7E. Please confirm whether you drew the images / clip-art within the figure panels by hand. If you did not draw the images, please provide (a) a link to the source of the images or icons and their license / terms of use; or (b) written permission from the copyright holder to publish the images or icons under our CC BY 4.0 license. Alternatively, you may replace the images with open-source alternatives. See these open-source resources you may use to replace images / clip-art:
-* [*https://commons.wikimedia.org*](https://commons.wikimedia.org) *-* [*https://openclipart.org/*](https://openclipart.org/)*.*

In response to your request regarding Figures 5A, 5C, 5D, 5E, 6A, 7A, 7C, and 7E, we confirm that all images and graphical elements included in these figure panels were entirely hand-drawn by the authors using design software Adobe Illustrator. The images were prepared by David Rojas as part of professional service. Therefore, there are no copyright restrictions on these illustrations.

*5) Thank you for stating "The datasets generated for this study can be found in the NIH repository under accession number PRJNA1234210 (*[*https://www.ncbi.nlm.nih.gov/sra/PRJNA1234210*](https://www.ncbi.nlm.nih.gov/sra/PRJNA1234210)*). This link reaches a 404 error page. Please amend this to a new link.*

We apologize for the error. The link has been amended. The link to the NIH repository (<https://www.ncbi.nlm.nih.gov/sra/PRJNA1234210>) will become active upon publication of the manuscript. Currently, the dataset is in a private status as per the repository's policy and will be made publicly accessible once the article is accepted for publication. However, we have generated a private link that allows reviewers to access the data during the review process:

<https://dataview.ncbi.nlm.nih.gov/object/PRJNA1234210?reviewer=c2tf8p4kt9epak4qe83rq5c6ia>

*6) Please ensure that the funders and grant numbers match between the Financial Disclosure field and the Funding Information tab in your submission form. Note that the funders must be provided in the same order in both places as well. Currently, these grants "PICT 2018-01494" and "PIP 201511220150100560CO" are missing from the Funding Information tab.*

We apologize for the omission. Grants "PICT 2018-01494" and "PIP 201511220150100560CO” have been incorporated in Funding Information tab.

7) Please provide a completed 'Competing Interests' statement, including any COIs declared by your co-authors. If you have no competing interests to declare, please state "The authors have declared that no competing interests exist". Otherwise please declare all competing interests beginning with the statement "I have read the journal's policy and the authors of this manuscript have the following competing interests:

As requested, we have included the exact required sentence in the manuscript text (line 819, Page 34).

**Answer to reviewers:**

**Reviewer #1, Overview.** *In this study, the authors use mice infected with Trypanosoma cruzi, the causative agent of Chagas disease, to examine cytotoxic CD8+ T follicular helper cells (Tfc), and in particular, their roles in influencing parasite-specific B cell responses. They report that Tfc promote B cell responses, while inhibiting plasmablast antibody production via Fas/FasL interactions, although this latter finding wasn’t restricted to Tfc, but also occurred in the presence of non-Tfc.*

**Reviewer #1.** *The* ***main issue*** *to address is making the images shown in Figures 1D & E more convincing. As it stands, I cannot see discrete CD8^+^ T cells in the germinal centres or surrounding plasmablasts.*

Using immunofluorescence on spleen sections, we aimed to demonstrate the presence of CD8⁺ cells within B cell follicles in *T. cruzi*-infected mice, and their absence in follicles from non-infected controls. In response to this observation, we enhanced the image quality in Figures 1D and 1E. Additionally, we included high-magnification insets in panel D for the 15 and 18 dpi samples, allowing clearer visualization of CD8⁺ cells within the B cell follicle. Accordingly, Figure 1 was reorganized, and the legend was updated (Lines 124–151, pages 5–7).

**Reviewer #1. Minor Issues:**

*Supp 1B: This figure indicates that non-Tfc includes non-CD8+ T cells (eg., CD4, B cells macrophages, etc.). If this is the case, I don’t think it is appropriate, as the comparison should be with non-Tfc CD8+ T cells. If the latter is the case, please change the Figure to reflect this, as indicated in the text (line 163).* Throughout the manuscript, the term Non-Tfc refers specifically to CD8⁺ T lymphocytes lacking CXCR5 and PD-1 expression, as shown in Figure 1. This population is composed exclusively of CD8⁺ T cells and does not include other cell types. In response to the reviewer’s comment, we have revised and corrected the legend of Supplementary Figure 1 (S1D Fig) to ensure precise identification of each cell population.

*Figure 1D & E: Better quality images are needed to show what is described in the text. For example, it is difficult to see CD8+ cells in the germinal centre (GC) at day 15 post-infection and determine if they are part of an extended peri-arteriol lymphoid sheath or represent distinct cells in the GC in Fig 1D.* As we indicated in our response to the **Major Issue**, we have modified the manuscript text in Results section to state that in *T. cruzi*-infected mice, CD8⁺ cells distributed throughout the B cell follicle, whereas these cells are absent in non-infected mice. Moreover, better quality images were included in Figure 1.

*Similarly, it is not clear there is co-localization between CD8 and CD138 at day 18 p.i., in Fig 1E.* We acknowledge that the original figure description may not have been sufficiently clear. We did not state that there was colocalization between CD8 and CD138, but rather indicated a close spatial proximity between these cell types. In response to the reviewer’s comment, we have revised the corresponding sentence in the Results section of the manuscript. In the updated version, we now specify that “In contrast to the follicular localization described above, immunofluorescence analysis at 18 and 23 dpi (Fig 1E) revealed that, outside B cell follicles, Ab-secreting cells identified by anti-CD138 staining (green) were found in proximity to CD8⁺ cells (red). These observations suggest potential spatial interactions between these two populations” (line 130-133, page 6). To improve visualization of this proximity, we also replaced the original 18 dpi image with a new one that better illustrates the proximity between CD8⁺ and CD138⁺ cells.

*Note: I could only access a pdf version of the Figures. It is not clear what the difference is between the upper and lower panels in Fig 1D at days 15, 18 and 23 p.i., and why two panels are shown for each time point.*

As we mentioned in the major issue, our objective was to highlight the presence of CD8⁺ cells within B cell follicles. Considering the reviewer’s observation, we decided to present only one of the spleen images with higher magnification view from animals infected at different dpi.

*Figure 2: Show the flow cytometry plots to indicate how cells were sorted (these can be Supplementary files).*

Flow cytometry plots to indicate how cells were sorted were included in S1B Fig. We have included this sentence to the manuscript: “S1B Fig illustrates the gating strategy employed for the sorting of Tfc and Non-Tfc CD8⁺ T cells.” (line 158-159, page 7).

*It is not clear why the authors refer to these cells as “cytotoxic” in the introduction, as the results in Fig 2C do not support this (i.e., IFNg and TNF up but granzymes down). This should at least be discussed.* The frequency of Granzyme B⁺ and Perforin⁺ cells is similar between Tfc and Non-Tfc populations in the spleens of *T. cruzi*-infected mice (Fig 4C). Tfc cells express both Granzyme B and Perforin and they have been previously described as cytotoxic (doi: 10.3389/fimmu.2022.1036616. PMID: 36591286). In response to the reviewer’s comment, we have included the following sentence in the Discussion section (line 544-546, page 23): “The frequency of Tfc cells expressing Granzyme B and Perforin is comparable to that observed in Non-Tfc cells (Fig 4C), suggesting that both subsets may exert cytotoxic.” Additionally, we incorporated the expression patterns of *Gzmb* and *Prf1* genes in the heatmap shown in Fig 2C.

*Figure 3: Show how CD4 Tfh cells were gated in Fig 3B.*

Following the reviewer’s suggestion, we have included the gating strategy used to identify Tfh cells in Supplementary Figure S1C, and this has been specified in the Results section (line 227, page 10).

*Representative flow plots should be shown for Fig 3C & D.*

The gating strategy shown in Fig 3A is the same one used for the analysis of the molecules presented in panels C and D. To clarify this point, we have modified the legend of Fig. 3 as follows: " Splenic cells from T. cruzi infected mice were collected at 18 dpi and analyzed by flow cytometry. (A) Representative contour plot (left) showing Tfc and Non-Tfc gating strategy, used for the analyses in panels A, C, and D."

In Result section, we included the following sentence: “The gating strategy shown in Fig 3A was used to define Tfc (CXCR5⁺PD-1⁺CD8⁺) and Non-Tfc (CXCR5⁻PD-1⁻ CD8⁺) populations. These gates were used for all subsequent phenotypic and functional analyses”. (Line 218-220, page 9).

*Figure 4: Show how Tfc and non-Tfc were gated in Fig 4A
Show gating used for Tfc and non-Tfc in Fig 4D.*

As indicated previously the gate strategy for Tfc and Non-Tfc was described in Fig 3A and detailed in S1B Fig.

*Figures 5-7: Clear experimental outline and execution.*
We appreciate the reviewer’ insightful observation.

**Discussion:**
*The authors should acknowledge limitations in their study, and in particular, short-comings in performing bulk RNAseq versus single cell RNAseq on antigen-specific Tfc cells (using available tetramer).* We thank the reviewer for this thoughtful comment. While we recognize the valuable insights that single-cell RNAseq of tetramer-positive Tfc cells could provide, particularly for exploring cellular heterogeneity and functional states, we considered bulk RNAseq to be the most appropriate approach for the objectives of this study. Our aim was to directly compare the transcriptional profiles of sorted Tfc and Non-Tfc CD8⁺ T cell subsets at the population level, and bulk RNAseq allowed us to achieve this effectively. We view these methodologies as complementary, each offering distinct advantages depending on the specific research question. In addition, single-cell RNAseq is not currently feasible in our setting due to the lack of local infrastructure. Although we explored the possibility of sending sorted cells abroad, we were unable to preserve cell viability adequately for transcriptomic analysis. These considerations have been taken into account in the interpretation and contextualization of our findings in the Discussion (lines 491-497, pages 20 and 21):

We chose to perform bulk RNAseq on sorted populations in order to directly compare the transcriptional profiles of Tfc and Non-Tfc cells at the population level, in line with the objectives of this study. Although single-cell RNAseq of antigen-specific Tfc cells could provide complementary information regarding heterogeneity and functional states, bulk RNAseq proved to be a robust and informative approach for defining the core transcriptional program of Tfc cells during *T. cruzi* infection.

*In addition, I feel the authors gloss over the cytotoxic features of the Tfc in T. cruzi infection. Their data indicates they are not as cytotoxic as in HIV or LCMV. Additionally, the potential role of TCF-1 expression is not discussed.* We agree that we did not extensively highlight the cytotoxic features of Tfc cells in the context of *T. cruzi* infection. As our study did not include direct cytotoxicity assays using *T. cruzi*-infected target cells, we are unable to draw definitive conclusions regarding their cytolytic capacity compared to Tfc cells described in HIV or LCMV infection models. Our primary aim was to explore the role of Tfc cells in supporting B cell responses.

Our data show that Tfc cells express Granzyme B and Perforin at levels comparable to Non-Tfc cells, suggesting a potential for cytotoxic activity. In the revised version of the manuscript, in Discussion section included a comment about this (line 544-546, page 23).

Regarding TCF-1, we have incorporated the following information into the discussion section (line 531-538, pag 22): Also, Tfc expressed higher levels of TCF-1, as evidenced by MFI determined by flow cytometry. TCF-1, encoded by *Tcf7*, is a key transcription factor involved in T cell development and in the differentiation and function programming of mature CD8⁺ T cells, including the generation of memory subsets (30). The elevated expression of TCF-1 observed in Tfc cells is consistent with the phenotype of this population detected in *T. cruzi* infected-mice, which includes central memory cells (30). In addition, TCF-1, along with Bcl6, appears to contribute to the transcriptional program underlying Tfc differentiation (42).

**Reviewer #2 Major Issues***: Although the authors have clearly demonstrated the functions of Tfc cells, it would be interesting, in a future experiment, to also investigate how Tfh cells assist B cells and whether the latter might have a regulatory role, given that they may also express Eomes, as shown for Tfc cells, and thus could potentially acquire this regulatory function.*

Following the reviewer’s suggestion, we decided to assess Eomes expression in Tfc cells and compare it with that of Tfh cells from *T. cruzi*-infected mice. In addition, we performed co-culture experiments with Tfh cells and either naïve B cells or plasmablasts to evaluate their helper and regulatory capacities, in comparison with Tfc cells.

Our analysis revealed that Tfh cells expressed Eomes; however, the expression levels were lower than those observed in Tfc cells. See histograms below. This information was not included in the new version of the manuscript

FMO

Tfc

Tfh

**Expression of Eomes in Tfh and Tfc cells**. Splenocytes from *T. cruzi*-infected mice were collected at 18 dpi and analyzed by flow cytometry. Representative histogram and quantification of Eomes MFI in Tfh (green) and Tfc (red) cells are shown. Data are presented as mean ± SD (N = 3 mice) from one independent experiment. Statistical analysis was performed using an unpaired *t*-test (*p* < 0.05, p < 0.01, *p* < 0.001, p < 0.0001).

The results from the co-culture experiments with Tfh cells and either naïve B cells or plasmablasts are shown in S5 Fig. We observed that Tfh cells (canonical B cell helper T cells), as expected, were capable of promoting Ab production by naïve B cells *in vitro*, even under short-term co-culture conditions (20 h), and to a similar extent as CD8⁺ Tfc cells, except for isotypes such as IgG2c and IgM. Moreover, when Tfh cells were co-cultured with differentiated B cells, such as plasmablasts, they can still modulate Ab production, although to a lesser extent than CD8⁺ Tfc cells.

The results have been incorporated into the Results section (lines 338-343 and 440-452, page 14, 18 and19), and in addition we have included a comment in the Discussion section of the new version of the manuscript (lines 566-571 and 591-596; pages 23-25).

*Another important point concerns CD107, which is more highly expressed in Tfc cells compared to non-Tfc cells. This is an important marker of degranulation and effector function. However, it is not clear whether B cells themselves could also be infected and targeted for destruction by these cells, or if their function is restricted solely to plasmablasts. Although the authors have demonstrated the role of Tfc cells during the acute phase of T. cruzi infection, and their expansion appears to correlate with parasitemia, it remains unclear whether these cells exert a more cytotoxic function during the chronic phase of the infection.*

We appreciate the reviewer’s insightful comment. Regarding the potential targets of Tfc cells, our co-culture experiments showed that plasmablast viability decreased in the presence of both Tfc and non-Tfc CD8⁺ T cells, whereas naïve B cells remained unaffected. This suggests that Tfc cytotoxicity is selective and likely depends on the activation or infection status of the target cell. Naïve B cells do not express Fas, making them resistant to Fas-dependent cytotoxic mechanisms, which may explain their survival. In contrast, plasmablasts and activated B cells upregulate Fas and may therefore be more susceptible to this pathway. Additionally, cytotoxic activity through degranulation would require that target cells be infected and present antigen via MHC I. A previous study showed that human B cells can be infected with *T. cruzi* (Doi: 10.1016/j.imbio.2020.151904), raising the possibility that infected B cells might be susceptible to CD8⁺ T cell-mediated killing. However, we did not evaluate this in our model and therefore cannot draw conclusions about the contribution of this pathway in our experimental system.

To explore whether Tfc cells exert cytotoxic functions beyond the acute phase, we analyzed their presence during the chronic stage of *T. cruzi* infection. Our data show that Tfc cells (CXCR5⁺PD-1⁺CD8⁺) are virtually absent at this stage, suggesting that their differentiation is restricted to the acute response or that they do not persist as such long term. While we cannot rule out the possibility that CXCR5⁺PD-1⁻ memory CD8⁺ T cells may derive from this population, the frequency of canonical Tfc cells during the chronic phase was too low to allow reliable functional analysis. Thus, the available data do not support a major cytotoxic role for Tfc cells during chronic infection, although this remains an interesting question for future studies. This information was not included in the manuscript.
Contour plots are shown below. We analyzed CXCR5 and PD1 expression on the CD8+CD4-B220- cell population.

Dot plots showing the expression of CXCR5 and PD-1 on CD8⁺CD4⁻B220⁻ lymphocytes from mice at 180 dpi.

**Part III – Minor Issues: Reviewer #2**: *The manuscript is very well written, with clearly defined objectives and a methodology that is appropriate and sufficiently rigorous to support the conclusions drawn.*

We appreciate the reviewer’s positive feedback and thoughtful comments.

*The authors employ a comprehensive experimental approach, ranging from transcriptomic analyses at different stages of infection to functional assays, which strengthens the validity of their findings. There are no major methodological flaws or ambiguous interpretations that would require clarification. The data are presented clearly and are well-supported by appropriate analyses.*

We sincerely appreciate the reviewer’s positive and stimulating feedback.

**Reviewer #3 Main issue***: Throughout the manuscript, the authors compare Tfc cells with non-Tfc cells, defined as CXCR5+PD-1+CD8+ and CXCR5-PD-1-CD8+ T cells, respectively. However, the gating strategies for these cell populations does not include any T cell activation markers (such as CD44), and as a result the non-Tfc population contains a significant proportion of naïve T cells (see Figure 4A). In fact, selecting PD-1- cells likely further enriches for naïve CD8+ T cells in the non-Tfc population. This issue complicates interpretations of phenotypic and functional differences between Tfc and non-Tfc cells, as the authors are essentially comparing an activated T cell population (Tfc) with a heterogenous population of T cells (non-Tfc) containing a large proportion of naïve, non-activated T cells, leading to some instances where the results are difficult to interpret. For instance, the RNA sequencing analysis indicates Tcf7 is expressed at higher levels in non-Tfc cells compared to Tfc cells, which contrasts with previously published results (PMIDs: 27487330, 27501248, 30377045). However, this result is likely due to the presence of a significant proportion of naïve CD8+ T cells (which highly express Tcf7) in the non-Tfc population. The authors should repeat analyses with the addition of T cell activation markers to identify both Tfc and non-Tfc cells. At the very least, the authors need to be more circumspect with their interpretations of comparative analyses between Tfc and non-Tfc cells to account for this issue.*

We thank the reviewer for this insightful comment. We agree that the Non-Tfc population, as defined in our gating strategy, comprises both naïve and activated cells as we show in Fig 4. However, our intention was not to imply that Tfc cells are the only CD8⁺ T cell subset capable of exerting cytotoxic or helper-like functions. Rather, we aimed to characterize the follicular CD8⁺ T cells (Tfc) and compare them to the broader Non-follicular CD8⁺ T cell compartment.

To address the reviewer concern, we evaluated the expression of key functional markers within the activated CD44⁺CD8^+^ T cells, thereby excluding naïve cells and focusing on activated CD8^+^ T cells that did not express CXCR5 or PD-1. Even when restricting the analysis to CD44⁺CD8^+^T cells, Tfc cells have higher expression of transcription factors (e.g., Bcl6), chemokine receptors (e.g., CXCR4), and helper-related molecules (e.g., CD40L), than activated CD44^+^ Non-Tfc (CXCR5^-^PD-1^-^CD8^+^) T cells.

We have included this experiment in the Results section, and it is presented as Supplementary Figure 3 (lines 261-269, page 11): “Given that Non-Tfc population contains a subset of naïve T cells, we reanalyzed the protein expression data shown in Fig 3 by excluding naïve CD8⁺ T cells. We then compared Tfc cells to CD44^+^ Non-Tfc cells (S3 Fig). Even after excluding naïve cells from the Non-follicular population, Tfc cells, all of which are CD44⁺, exhibited higher levels of ICOS, CD40L, Bcl6, CXCR3, CXCR4, CCR7, T-bet, Eomes, and TCF-1 compared to CD44⁺ Non-Tfc cells. Taken together, the higher expression of those proteins in Tfc from *T. cruzi*-infected mice is not related to the activation status of CD8⁺ T cells, since activated CD44⁺ Non-Tfc CD8⁺T cells expressed significantly lower levels of these molecules compared to Tfc cells.” Moreover, we have included this statement in the Discussion section: “Tfc cells may represent a distinct and functional CD8⁺ T cell population that emerges during *T. cruzi* infection, characterized by features not merely attributable to an activated state”. (line 539-541, page 22).

**Reviewer #3: Minor Issues:**

• *For all data presented, the number of biological and/or technical replicates and the number of independent experiments performed for each analysis should be clearly indicated for each dataset. This is currently difficult to determine from some of the figure legends.*

In response to the reviewer’s comments, we carefully reviewed all figure legends and have incorporated the requested information.

• *It appears the data in Figure 5B are used again in Figure 5D. Were these experiments conducted separately, as indicated in the figure legend, or at the same time? If the data are from the same experiment this should be clearly communicated in the figure legend.*

We appreciate the reviewer’s observation. Considering that the experiments were performed more than once, and in order to avoid redundancy and ensure clarity, we have incorporated the result of an independent experiment (immunoglobulin determination), different from the one shown in Fig. 5B, but with similar data that lead to the same conclusion.

*• The authors identify a role for Tfc cells in promoting IgG2c production by B cells in in vitro co-cultures. A previous publication looking at the role of CXCR5+CD8+ T cells in shaping antibody responses to immunisation and peripheral viral infection has demonstrated these cells promote IgG2c responses in vivo (PMID: 34326833). This study should be referenced in this manuscript.*

We thank the reviewer for this helpful suggestion. We have now cited the study by Tyllis et al. (PMID: 34326833) in the Discussion section of the revised manuscript, as follows: “Consistent with our findings, Tyllis et al. (60) reported that CXCR5⁺CD8⁺ T cells can promote IgG2c responses *in vivo*, further highlighting the potential role of Tfc cells in regulating isotype switching and shaping humoral immunity.”. (line 564-566, page 23).

• *Line 149, the anatomical compartment (presumably blood?) in which parasitemia is assessed should be included in the figure legend. Additionally, the assay for determining parasitemia should be described in the methods.*

We have indicated in Figure 1 legend (line 143, page 6) that parasitemia (parasites count in blood) was assessed in blood samples. Additionally, the assay used to determine parasitemia has been described in the Methods section (lines 630-632, page 26) of the revised version of the manuscript.

*• Line 157, the authors should be specific as to which data in the figure are from three independent experiments.*

As requested, we have clarified in the Figure 1 legend that the kinetic data (Figure 1A–C) originate from two independent experiments, while the immunofluorescence data (Figure 1D–E) were obtained from three independent experiments.

*• Line 183, Mki67 is not a transcription factor encoding gene*…

We revised the sentence in the manuscript and replaced *"transcription factors"* by *"genes,"* since some of the included markers, such as **Mki67** and **mTOR**, are not transcription factors. Similarly, in the heatmap, we updated the label *"transcription factors"* to better represent gene categories associated with differentiation, proliferation, and cell activation.

*• Line 202, include time point (18-dpi?) of analysis in the figure legend.*

We have now included the time point in the Figure 2 legend. As indicated, RNA-seq analysis was performed on cells collected at 18 dpi.

*• Line 223, how was CD40L measured, intracellular or surface staining? This should be detailed in the figure legend and methods section.*

CD40L expression was determined by surface staining. This information was detailed in the legend (lines 244-245, page 10) and in the Methods section (lines 662-663, page 27).

*• Lines 276-278, a reference should be provided to support this conclusion.*

We have added three references, detailed below, that support our conclusion that effector T cells exhibit higher glucose uptake than non-effector T cells. Additionally, we included another reference to support the methodology used. (lines 286-288, page 12)

31.	Chang CH, Curtis JD, Maggi LB, Faubert B, Villarino AV, O’Sullivan D, et al. Posttranscriptional Control of T Cell Effector Function by Aerobic Glycolysis. Cell. 2013 Jun 6;153(6):1239–51. doi: 10.1016/j.cell.2013.05.016

32.	MacIver NJ, Michalek RD, Rathmell JC. Metabolic regulation of T lymphocytes. *Annu Rev Immunol*. 2013;31:259-283. doi:10.1146/annurev-immunol-032712-095956

33.	Ana Y, Rojas Marquez JD, Fozzatti L, Baigorrí RE, Marin C, Maletto BA, et al. An exacerbated metabolism and mitochondrial reactive oxygen species contribute to mitochondrial alterations and apoptosis in CD4 T cells during the acute phase of Trypanosoma cruzi infection. Free Radic Biol Med. 2021 Feb 1;163:268–80. doi: 10.1016/j.freeradbiomed.2020.12.009

*• Line 317, IgM is not a class-switched Ig.*

We thank the reviewer for pointing out this inadvertent mistake. The original sentence was modified as “A multiplex bead-based assay demonstrated that Tfc cells promoted the secretion of IgM and class-switched Igs, including IgA, IgG2b, IgG2c, and IgG3 (Fig 5B)”. (line 326, page 14).

*• The experiment in Figure 5E should be performed with an isotype control antibody in the control cultures.*

The experiment included an isotype control antibody, as indicated in the legend of Fig. 5 (Line 373, Page 16 - Fig 5E) and in the Methods section. Details of all antibodies used are provided in the Methods section (Lines 733-735, Page 30).

• Line 359, what is the assigned value below the LOD? Is it zero?

To enable statistical analysis, undetectable measurements were assigned a value equal to the lower limit of detection (LOD). The LOD varies depending on the specific immunoglobulin and is determined from the standard curve generated in each experimental run. This has been clarified in the manuscript with the following sentence: “For statistical analysis, samples with non-detectable (ND) values were imputed with a value equal to the lower detection limit of the technique employed.” (lines 374-375, page 16).

• Lines 482-486, references should be provided to support each point in this sentence.

The requested references have already been included in the text (lines 520-521, page 22):

47.	Standiford TJ, Kunkel SL, Greenberger MJ, Laichalk LL, Strieter RM. Expression and regulation of chemokines in bacterial pneumonia. J Leukoc Biol. 1996 Jan 1;59(1):24–8. doi: 10.1002/jlb.59.1.24

48.	Liu M, Li L, Cao L, Li W, Gu X, Yang M, et al. Targeted delivery of CCL3 reprograms macrophage antigen presentation and enhances the efficacy of immune checkpoint blockade therapy in hepatocellular carcinoma. J Immunother Cancer. 2025 Feb;13(2):e010947. doi: 10.1136/jitc-2024-010947

49.	Lindell DM, Standiford TJ, Mancuso P, Leshen ZJ, Huffnagle GB. Macrophage Inflammatory Protein 1α/CCL3 Is Required for Clearance of an Acute Klebsiella pneumoniae Pulmonary Infection. Infect Immun. 2001 Oct;69(10):6364–9. doi: 10.1128/IAI.69.10.6364-6369.2001

50.	Raza S, Rajak S, Tewari A, Gupta P, Chattopadhyay N, Sinha RA, et al. Multifaceted role of chemokines in solid tumors: From biology to therapy. Semin Cancer Biol. 2022 Jan 1;S1044-579X(21)00304-7. doi: 10.1016/j.semcancer.2021.12.011

51.	Huang H, Li F, Cairns CM, Gordon JR, Xiang J. Neutrophils and B Cells Express XCR1 Receptor and Chemotactically Respond to Lymphotactin. Biochem Biophys Res Commun. 2001 Feb 23;281(2):378–82. doi: 10.1006/bbrc.2001.4363.

*• Line 511, what is meant by ‘fully’ naïve B cells?*

We used the term 'fully naïve' to highlight that these B cells were entirely antigen-inexperienced, maintaining the phenotype of cells newly released from the spleen. Nevertheless, we acknowledge that the qualifier 'fully' is unnecessary and could be misleading. Accordingly, 'fully' it has been removed from the text.

• *Line 531, the conclusion that this reports the first evidence of direct modulation of plasmablast viability by Tfc cells requires more nuance as this capability is not unique to Tfc cells in T cruzi infection. As identified in this study, non-Tfc cells appear to be similarly able to kill plasmablasts ex vivo.*

We agree with the reviewer that both Tfc and Non-Tfc cells exhibit similar capacity to kill plasmablasts ex vivo. In our conclusion, our intention was to emphasize that this is the first study to describe a role for Tfc cells in modulating plasmablast viability—an aspect not previously reported. We have revised the conclusion as follows: “our results provide the first evidence of direct modulation of plasmablast viability by CD8⁺ T cells during T. cruzi infection, a function mediated by Tfc cells and also detectable in Non-Tfc cells”. (line 585-587, page 24).

• *All flow cytometry plots should be presented as pseudocolour dot plots or contoured plots with outliers, not as density plots*.

All density plots, including those from supplementary figures, were changed to contoured plots.

*• Axes for flow plots should also include fluorophore labels.*

Fluorophore labels have been included in all flow plots.

• *Given naïve cells are present in the non-Tfc population, what level of confidence do the authors have that the flow cytometry data accurately reflect protein expression of CCR7 and TCF-1 as presented in Figure 3 C and D. The flow cytometry appears to contradict the RNA sequencing results.*

As shown in the new S3Fig, even when naive T cells were excluded from flow cytometry analysis, Tfc cells exhibited higher levels of CCR7 and TCF-1 protein compared to activated Non-Tfc CD8^+^ T cells. We acknowledge the discrepancy between these protein expression data and our RNA-seq results, which showed lower *Ccr7* and *Tcf7* transcript levels in Tfc cells. This inconsistency may be explained by post-transcriptional regulation mechanisms (doi: 10.1042/BST20150154, ref. 44), which can result in a lack of direct correlation between mRNA and protein expression levels. We have added a sentence to the Discussion section to address this point (line 508-509, page 21).

• *Line 532, the authors content that due to the lack of tolerance checkpoints for plasmablasts their elimination by Tfc cells may contribute to regulation of humoral immunity. However, this statement is not consistent with published results. In T cruzi infection, this group has previously shown that plasmablast responses in this model are dependent on a checkpoint involving BCL6-expressing CD4+ T cells (PMID: 35651611).*

Thank you for pointing this out. We have revised and modified the statement accordingly and incorporated this point into the Discussion section (lines 588-602, pages 24 and 25): “Our group has previously demonstrated that CD4⁺ Tfh cells expressing Bcl6 are essential for the generation of extrafollicular plasmablast during *T. cruzi* infection (7). In the current study, we confirmed that Tfh cells, as expected, promoted Ab production by naïve B cells *in vitro*, even under short-term co-culture conditions (20 h), and to a similar extent as CD8⁺ Tfc cells, except for IgG2c and IgM. However, when co-cultured with differentiated B cells such as plasmablasts, Tfh cells reduced Ab production less efficiently than Tfc cells. Together, these findings support the idea that while CD4⁺ Tfh cells are critical for the initiation of the plasmablast response, CD8⁺ Tfc cells may act at a later stage to eliminate differentiated plasmablasts. Thus, Tfc-mediated killing could provide an additional mechanism of regulation to restrain potentially harmful humoral responses during infection. CD8⁺ Non-Tfc cells were also able to control plasmablast viability. This suggests that both Tfc and Non-Tfc populations might contribute to the control of extrafollicular B cell responses, particularly targeting plasmablasts that have not undergone the same stringent selection mechanisms as germinal center B cells (65,66).”

*• The manuscript would benefit from a thorough grammatical review, especially the methods section and reference list.*

We have thoroughly checked the grammar throughout the manuscript, with special emphasis on methods section and reference list. Since there are several editing issues, we have not marked it in red unless new information has been added.

*• Line 565, presumably 70 μm (not 0.70 μm) cell strainers were used in cell preparation?*

Yes, we have used 70 um cell strainers. This information was corrected in the text (line 636, page 26).

*• The link to the RNA sequencing data set does not seem to work. This should be rectified before publication*.

You can view the uploaded files at the following link: <https://dataview.ncbi.nlm.nih.gov/object/PRJNA1234210?reviewer=c2tf8p4kt9epak4qe83rq5c6ia> . This link (<https://www.ncbi.nlm.nih.gov/sra/PRJNA1234210>) will be valid once the paper is published.

*• In figure 4B and 4C, have the authors assessed viability of B cells in co-cultures where CD8+ T cells are stimulated with anti-CD3/anti-CD28 or TSKB20? If so, are there any difference in B cell viability between Tfc and non-Tfc co-cultures in the anti-CD3/anti-CD28 or TSKB20 stimulation conditions?* *This may be interesting as TSKB20 would likely be presented on MHC-I on B cells in this setting, leading to cognate interactions between CD8+ T cell populations and B cells.*

We assessed B cell viability under anti-CD3/anti-CD28 or TSKB20 stimulation and found no differences in the frequency of live B cells between the two stimuli when co-cultured with either Tfc or Non-Tfc cells.

• *Have the authors assessed ICOSL expression on CXCR5+CD8+ T cells in T cruzi infection? This should be assessed considering ICOSL is expressed by regulatory CXCR5+CD8+ T cells in some contexts (PMID: 20844537)*.

We evaluated ICOSL expression by flow cytometry in Tfc and Non-Tfc cells. Based on FMO controls, neither population expressed detectable levels of ICOSL. This result was not included in the manuscript, as it did not show differences between Tfc and non-Tfc cells and we chose to focus on phenotypic markers more relevant to the study.

**Expression of ICOSL in Tfc and Non-Tfc cells**. Splenocytes from *T. cruzi*-infected mice were collected at 18 dpi and analyzed by flow cytometry. Representative histogram of ICOSL MFI in Tfc (red) and Non-Tfc (blue) cells. The fluorescence minus one (FMO) control is shown in gray.

• *Do B cells with neutralising specificities become infected with T cruzi, resulting in their cytotoxic elimination by CD8+ T cells and a delayed onset of neutralising serum antibody titres, as is the case in LCMV infection (PMID: 27812556).*

We have not determined whether B cells specific for *T. cruzi* antigens become infected. This is a plausible scenario and would be an interesting aspect to investigate. However, it would require studies using electron microscopy and the availability of a mouse model in which antigen-specific B cells are suitably labeled. At this stage, we cannot provide any conclusions on this matter, as it would be highly speculative.

• *The RNA sequencing analysis indicates that several genes involved in MHC-II antigen processing and presentation (H2-Aa, CD74, H2-Eb1, H2-Ab1, Ciita) are upregulated in Tfc cells compared to non-Tfc cells. In the methods section the authors note that Tfc cells are sorted by negativity for B220 (to exclude B cells), but this result suggests some B cell contamination may be present in the Tfc population. What are the read counts for these genes in Tfc cells? What was the sort purity for the cell populations in this analysis*? *The authors should comment on this result.*

Below, sort purity of each subset is shown:

Tfc

Non-Tfc

**Sort purity of Tfc and Non-Tfc cells post cell sorting**. Representative flow cytometry plots showing gating strategy for Tfc and Non-Tfc cells identification.
Lymphocytes were gated based on FSC-A vs. SSC-A parameters (top and bottom rows, respectively). Middle panels: CD8⁺ T cells were identified within the CD4⁻B220⁻ population showing a purity higher 95%. Right panels: Expression of CXCR5 and PD-1 was analyzed within the CD8⁺ T cell population to define follicular cytotoxic CD8⁺ T cells (Tfc; top, red) and non-follicular CD8⁺ T cells (Non-Tfc; bottom, blue).

Although transcripts associated with MHC class II antigen presentation (such *as H2-Aa, CD74, H2-Eb1, H2-Ab1,* and *Ciita*) were detected in the Tfc RNA-seq dataset, this does not necessarily indicate contamination with B cells. Post-transcriptional regulation can result in low or absent protein expression despite detectable mRNA levels. To evaluate this, we assessed MHC class II surface expression by flow cytometry in sorted Tfc cells and compared it to naïve B cells as a positive control. Tfc cells showed low but detectable expression of MHC class II, markedly lower than B cells, suggesting that they may upregulate some components of the MHC-II pathway without expressing these molecules at high levels on the surface. This result was not included in the manuscript, as it did not alter the main conclusions of the study.

**Expression of MHC II in Tfc and B cells.** Tfc and naïve B cells were sorted from splenocytes of *T. cruzi*-infected mice at 18 days post-infection (dpi) and analyzed by flow cytometry. Representative histograms show MHC II expression in sorted Tfc (red; CXCR5⁺PD-1⁻CD8⁺) and naïve B cells (IgD⁺B220⁺). The fluorescence minus one (FMO) control is shown in gray.
