# Supplementary material for: Follicular CD8+ T cells in Trypanosoma cruzi infection: helpers or killers depending on the target B cell population
Source: PLoS Pathog. 2025 Oct 17;21(10):e1013595. doi: 10.1371/journal.ppat.1013595 (PMC12558609; doi:10.1371/journal.ppat.1013595)
Supplement: Attachment — Submitted filename: Response_to_Reviewers_auresp_2.docx [file ppat.1013595.s009.docx]

Córdoba, Argentina, October 1st, 2025

Martin Craig Taylor
Guest Editor-PLOS Pathogens

Thanks for your correspondence regarding our manuscript PPATHOGENS-D-25-00863, “Follicular CD8⁺ T cells in Trypanosoma cruzi infection: helpers or killers depending on the target B cell population”, in which you conveyed the request from Reviewer #3.

We would also like to thank the reviewer for their thoughtful and constructive comments on our manuscript. We have incorporated the requested information and clarified the text accordingly.

Below, we provide a detailed response to the reviewer’s comments. In addition, the requested information has been included in the Results, Materials and Methods, and in the legend of supplementary Figure 3 in the new version of the manuscript. We hope that the reviewer will find our responses satisfactory.

Along with this rebuttal letter addressing each of the reviewer’s points, we have uploaded a marked-up copy of our manuscript highlighting the changes made to the original version, labeled “Revised Manuscript with Track Changes” . We have also uploaded a clean version of the revised paper without tracked changes, labeled “Manuscript.”

We look forward to hearing from you soon.

Sincerely,

**Adriana Gruppi**

Centro de Investigaciones en Bioquímica Clínica e Inmunología.

Facultad de Ciencias Químicas. Universidad Nacional de Córdoba

**Answer to reviewer # 3**

Part III – Minor Issues

Reviewer #3: -Density plots in figure 6B still require updating to contoured dot plots.

We apologize for this unintentional mistake. We followed the reviewer’s initial suggestions but unfortunately we uploaded an outdated version of the figure. We have now carefully reviewed all submitted material and are providing the corrected figure, in which the density plots in Figure 6B have been replaced with contoured dot plots.

-A few axes for flow plots still require updating with fluorophore labels (for instance: figure 6B, 7D). In figure 3A, for the fluorophore label for CXCR5 on the y-axis, just show which fluorophore is in the representative plot (not both PECy7 and APC).

In accordance with the reviewer’s observation, we have corrected the mentioned figures. The fluorophore labels have been added in Figures 6B and 7D. In Figure 3A, the fluorophore label for CXCR5 on the y-axis has been specified as PECy7, which is the most representative.

-Naive CD8 T cells express CCR7 and TCF-1 and since there are naive CD8+ T cells present in the non-Tfc population, the absence of clear CCR7 and TCF-1 positive populations in non-Tfc cells in fig 3C-D is concerning. Have the authors performed CCR7 staining under specific conditions (after resting cells or staining at 37 degrees) which are necessary for CCR7 staining?

We have performed CCR7 staining at 37 degrees. In the new version of the figure, we included the results obtained at 37 degrees. This information has been included in the Materials and Methods section (Line 680-681, Page 28).

The authors should compare CCR7 and TCF-1 protein expression in naive CD8+ T cells from non-infected and infected mice to assess whether the apparent absence of CCR7 and TCF-1 protein expression in non-Tfc cells observed here is a result of T cruzi infection or any potential issues with staining these molecules. While I note the response to my previous comment on this issue and data presented in fig S3B, this still needs to be resolved as the absence of CCR7 and TCF-1 from non-Tfc cells (which contain naive CD8+ T cells) is irregular.

We appreciate the reviewer’s comment. Conducting CCR7 staining at 37 °C resulted in a clear improvement in staining quality. We measured CCR7 and TCF-1 on naïve CD8⁺ T cells from non-infected mice (Tfc are absent in non-infected mice), as well as on Tfc and Non-Tfc cells from infected mice. In non-infected mice, a clear fraction of naïve CD8⁺ T cells expressed both molecules (CCR7 and TCF-1), confirming expected result. In contrast, within the Non-Tfc population of infected mice, which includes a proportion of naïve T cells, the frequency and MFI of CCR7⁺ and TCF-1⁺ cells were markedly reduced compared with non-infected controls. This reduction is most likely a consequence of *T. cruzi* infection, as the reviewer suggested, rather than a technical issue with staining. Notably, within the Tfc population we detected a lower frequency of CCR7⁺ and TCF-1⁺ cells, although CCR7 MFI was slightly increased and TCF-1 levels remained unchanged relative to naïve CD8⁺ T cells from non-infected mice.

The expression of CCR7 and TCF-1 observed within the Tfc population could be explained by the presence of central memory CD8⁺ T cells, as these markers are characteristic of this subset.

We have added histograms and bar graphs presenting CCR7 and TCF-1 data from CD8^+^ T cells from non-infected and infected mice in S3E Fig. Furthermore, Figure 3C has been updated to provide an improved graph of CCR7 expression in non-Tfc cells, where the positive subset can now be more clearly appreciated.

Accordingly, in Result Section we modified the text as follows (lines 270-279, pages 11-12):

“We showed that CCR7 expression was reduced in Non-Tfc cells from infected mice, despite this population including a subset of naïve CD8⁺ T cells that typically express high levels of CCR7 and TCF-1 (31,32). To evaluate whether this pattern could be explained by infection, we compared CCR7 and TCF-1 expressions in Tfc and Non-Tfc cells from infected mice with that in naïve CD8⁺ T cells from non-infected controls. As shown in S3E Fig, naïve CD8⁺ T cells from non-infected mice (CD8^+^ naïve NI) expressed CCR7 and TCF-1, and this expression was higher than in Non-Tfc cells from infected mice. Within the Tfc subset, the frequency of CCR7⁺ and TCF-1⁺ cells was also reduced; however, CCR7 MFI was slightly higher than that observed in naïve CD8⁺ T cells from non-infected mice, while TCF-1 levels remained comparable.”

The legend of S3 Fig has been revised (lines 1146-1160, page 48-49).

Based on these results in Discussion Section we included the following text (Line 549-555, Page 23):

“It is interesting to note that CCR7 and TCF-1 expression was reduced in Non-Tfc cells from infected mice compared with naïve CD8⁺ T cells from non-infected mice, even though Non-Tfc cells still include some naïve CD8⁺ T cells. This reduction likely reflects infection-induced modulation of chemokine receptors, which may result from prolonged exposure to their ligands, potentially increased due to pathogen-driven inflammation, or could represent a pathogen strategy to prevent the migration of cells involved in host defense (60–62).”
